# Impact of mobile health technologies on human papillomavirus vaccination uptake among mothers of unvaccinated girls aged 9–14 years in Lagos, Nigeria (*mHealth-HPVac*): study protocol of a randomised controlled trial

**DOI:** 10.1186/s12885-024-12538-6

**Published:** 2024-06-20

**Authors:** Kehinde S. Okunade, Adebola A. Adejimi, Temitope V. Adekanye, Matthew J. Allsop, Hameed Adelabu, Olufemi Thomas-Ogodo, Tonia C. Onyeka, Teniola Lawanson, Godwin O. Akaba, Omolola Salako, Rose I. Anorlu, Jonathan S. Berek

**Affiliations:** 1 Department of Obstetrics and Gynaecology, College of Medicine, University of Lagos, Lagos University Teaching Hospital, PMB 12003, Surulere, Lagos, Nigeria; 2 Department of Obstetrics and Gynaecology, Lagos University Teaching Hospital, Surulere, Lagos, Nigeria; 3 Department of Community Health and Primary Care, College of Medicine, University of Lagos, Surulere, Lagos, Nigeria; 4 Academic Unit of Palliative Care, Leeds Institute of Health Sciences, University of Leeds, Leeds, UK; 5 Center for Clinical Trials, Research, and Implementation Science, (CCTRIS), College of Medicine, University of Lagos, Surulere, Lagos, Nigeria; 6 Department of Anaesthesia, College of Medicine, University of Nigeria, Enugu, Enugu State Nigeria; 7 Department of Obstetrics and Gynaecology, College of Health Sciences, University of Abuja, Gwadalada, Abuja FCT Nigeria; 8 Department of Radiation Biology, Radio-diagnosis and Radiography, College of Medicine, University of Lagos, Radiotherapy, Surulere, Lagos, Nigeria; 9 Department of Obstetrics and Gynaecology, Stanford University School of Medicine, Stanford, Palo Alto, CA USA

**Keywords:** Cervical cancer, Lagos, Mhealth-cervix, NHIS, Nigeria, Pap test

## Abstract

**Background:**

Despite the availability of effective vaccines, human papillomavirus (HPV) vaccine uptake remains low in most resource-limited settings including Nigeria. Mobile health technology (mHealth) has the potential to empower patients to manage their health, reduce health disparities, and enhance the uptake of HPV vaccination.

**Aim:**

The “*mHealth-HPVac*” study will assess the effects of mHealth using short text messages on the uptake of HPV vaccination among mothers of unvaccinated girls aged 9–14 years and also determine the factors influencing the uptake of HPV vaccination among these mothers.

**Methods:**

This protocol highlights a randomised controlled trial involving women aged 25–65 years who will be enrolled on attendance for routine care at the General Outpatient clinics of Lagos University Teaching Hospital, Lagos, Nigeria between July and December 2024. At baseline, *n* = 123 women will be randomised to either a short text message or usual care (control) arm. The primary outcome is vaccination of the participant’s school-age girl(s) at any time during the 6 months of follow-up. The associations between any two groups of continuous variables will be assessed using the independent sample t-test for normally distributed data, or the Mann-Whitney U test for skewed data. For two groups of categorical variables, the Chi-square (*X2*) test or Fisher’s exact test will be used, as appropriate. Using the multivariable binary logistic regression model, we will examine the effects of all relevant sociodemographic and clinical variables on HPV vaccination uptake among mothers of unvaccinated but vaccine-eligible school-age girls. Statistical significance will be reported as *P* < 0.05.

**Discussion:**

The mHealth-Cervix study will evaluate the impact of mobile technologies on HPV vaccination uptake among mothers of unvaccinated but vaccine-eligible school-age girls in Lagos, Nigeria as a way of contributing to the reduction in the wide disparities in cervical cancer incidence through primary prevention facilitated using health promotion to improve HPV vaccination uptake.

**Registration:**

PACTR202406727470443 (6th June 2024).

**Supplementary Information:**

The online version contains supplementary material available at 10.1186/s12885-024-12538-6.

## Introduction

In 2020, there were an estimated 604,000 new cases of cervical cancer and 342,000 deaths from cervical cancer, with 70% of these deaths occurring in developing countries [1]. Persistent infection of the cervix with certain high-risk genital human papillomavirus (HPV) is a necessary cause of cervical cancer [2, 3]. One of the most effective strategies for cervical cancer prevention is vaccination against HPV infection among young and adolescent girls before the start of sexual activity [2, 4].

Administering HPV vaccines in low and middle-income countries is essential for achieving the global action plan aimed at bridging the cervical cancer gap [5]. HPV vaccine rollout to girls aged 9–14 years as recommended by the World Health Organization (WHO) [6] is projected to have a greater and faster direct impact and herd effects on the population’s immunity [7]. There are four vaccines against HPV infections [8, 9] and only two of these (quadrivalent Gardasil and bivalent Cervarix) are currently approved for use by the Nigerian Federal Ministry of Health [10, 11]. In October 2023, Nigeria embarked on new routine HPV vaccine roll-out campaigns to reach about 7.7 million girls [12]. Under this new immunization protocol, girls aged 9 to 14 years will receive a single dose of the vaccine [12], which is highly effective at preventing infection of HPV types 16 and 18 that are known to cause at least 70 per cent of cervical cancers [2].

Despite the availability of effective vaccines, HPV vaccine uptake remains low in most resource-limited settings including Nigeria [5, 12, 13]. The utilization of mobile technologies has increased significantly in recent years [14] with increased opportunities for mobile health technologies (mHealth) development [15]. Mobile health technology (mHealth) may empower patients to control their health, reduce inequalities [16], and improve the uptake of health interventions such as HPV vaccination. There are only a few reported studies in Sub-Saharan Africa that examined the use of mHealth in cancer prevention [16] but there are currently none that have investigated the impact of this intervention on the uptake of HPV vaccination among mothers of eligible unvaccinated vaccine-eligible school-age girls.

The primary objective of this proposed study “*mHealth-HPVac*”, therefore, is to assess the effects of mHealth using short text messages on the uptake of HPV vaccination among mothers of unvaccinated girls 9–14 years while the secondary objective is to determine the factors affecting the uptake of HPV vaccination among mothers of unvaccinated girls aged 9–14 years under usual conditions. This is innovative because the introduction of mHealth, now regarded as one of the most promising investments for health in developing countries [17], is a novel concept in the paradigm shift for disease prevention which can contribute to improving cervical cancer control in the resource-limited settings of sub-Saharan Africa (SSA). Thus, the study will generate the first real-world evidence in SSA on the efficacy of mobile technologies on HPV vaccination uptake among eligible school-age daughters of participating mothers in Nigeria.

## Methodology

### Study design and setting

*“mHealth-HPVac”* is a randomised parallel arm controlled trial of mothers of unvaccinated girls aged 9–14 years who attend routine care at the General Outpatient (GOP) clinics of the Lagos University Teaching Hospital (LUTH), Nigeria between June and September 2024. LUTH is the leading healthcare institution in Lagos, acting as a referral centre for other public and private hospitals in Lagos and nearby states of Ogun and Oyo. The hospital offers a range of services, including comprehensive gynaecologic oncology prevention such as Pap smears, human papillomavirus testing, colposcopy, and pathological services, including cytology and histology [18]. The GOP clinic of the hospital is opened on each day of the week with attendees being mainly enrollees of the National Health Insurance Scheme.

### Study population

Eligible participants are mothers of unvaccinated girls aged 9–14 years; who express willingness to vaccinate their children; own and use a personal cellphone; free from any mental or physical disabilities that inhibit them from understanding the implications of the study and not considering relocating from their current residence within the next year. The exclusion criteria include refusal of consent or withdrawal of consent during the study.

### Study endpoints and sample size calculation

The *primary endpoint* is the prevalence of single-dose HPV vaccination uptake after 6 months and the *secondary endpoints* are predictors of HPV vaccination uptake after 6 months of enrolment among participating mothers of young or adolescent girls aged 9–14 years. Participants will be tracked via medical record review as well as through phone calls after the 6th month of enrolment to collect data on their HPV vaccination uptake. With pooled HPV vaccine uptake prevalence of 28.5% for a usual care condition derived from the systematic review by Asgedom et al [19] and an expected attrition rate of 20%, a sample size [20] of *n* = 123 women is expected to provide 80% power to establish a 30% proportional difference in HPV vaccination uptake between mHealth intervention and usual care condition.

### Participants’ enrolment and data collection

**Fig. 1:**
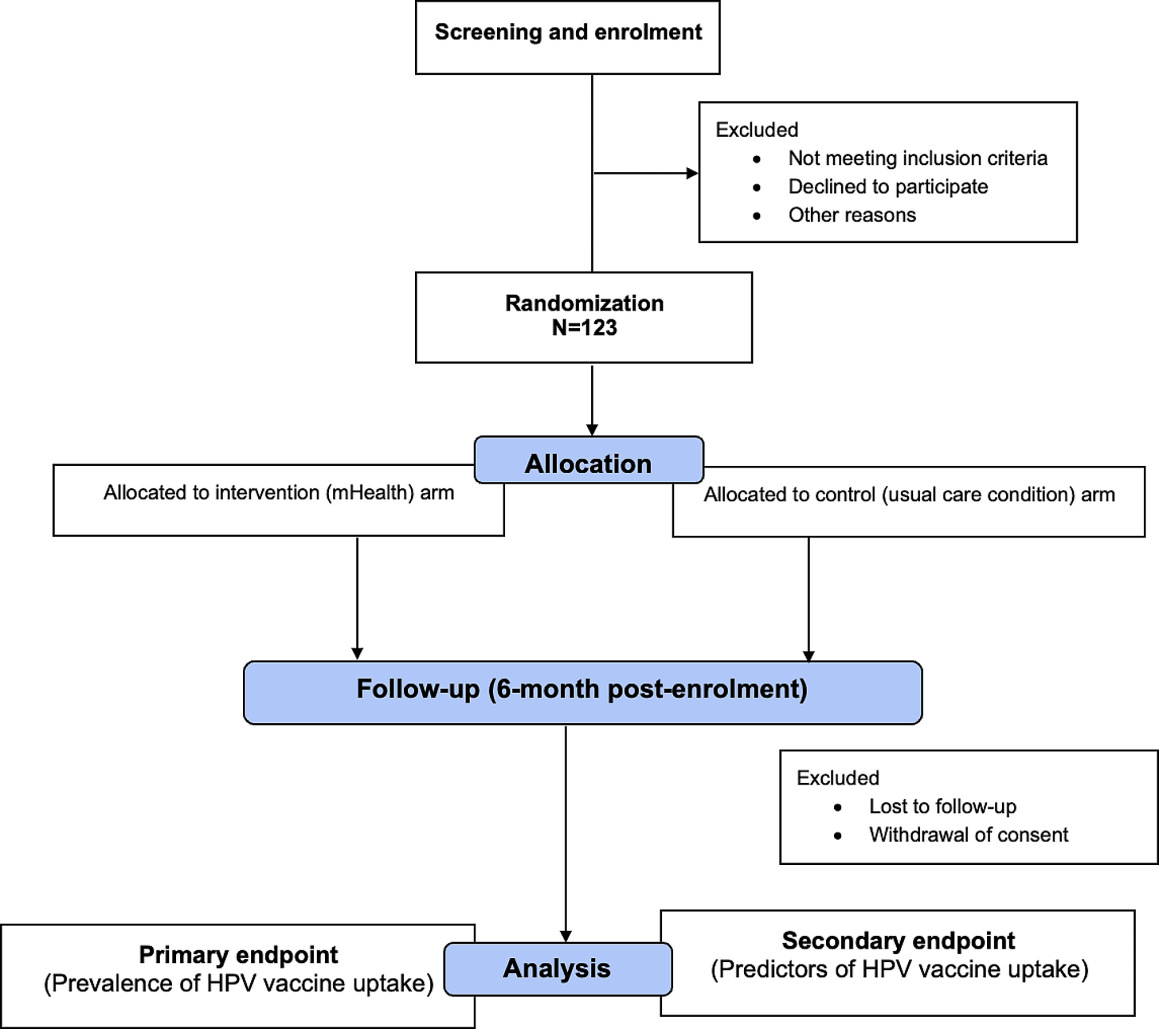
Trial flow chart

A 20–30-minute health talk on cervical cancer prevention including HPV vaccination is given by the clinic midwives as part of usual care to all women in the clinics after which the investigators or research assistants will screen and identify eligible women for the study [Fig. 1]. The women will then be invited to give consent for participation upon explanation of the purpose and procedures of the study. Once consent is obtained, an electronic interviewer-administered questionnaire created on the REDCap database will be applied to each participant to obtain baseline information on sociodemographic variables, cellphone use and distance of participants’ residence from the clinics (measured in kilometres using Google map).

### Randomization and allocation concealment

Once enrolled, eligible women will be randomised to either a text message (intervention) arm or a usual care (control) arm using a computer-generated random sequence generated by an independent statistician.

- *Intervention (mHealth) arm* – MultiTexter Bulk short message service (SMS) will be used as the platform to deliver the mHealth messages given its reliability and low cost. Participants will be sent messages containing information on cervical cancer and are then encouraged to take their daughters aged 9–14 years for HPV vaccination. Text messages will be delivered monthly for 6 months after enrollment.
- *Usual care (control) arm* – Participants in this study arm will only receive the usual health education talk at enrolment. They will receive no additional follow-up text messages.

Group allocations will be concealed from the investigators and trial statistician using sealed opaque envelopes that are sequentially numbered. The envelopes will be prepared in advance of the trial and will only be opened sequentially at the time of participants’ enrollment, ensuring that the treatment allocation remains concealed until the point of assignment. Given that no safety concerns are expected, we do not anticipate the need for unblinding during the study.

### Data management and statistical analysis

Data collection will occur prospectively with data entry and checking taking place continuously. Queries will be actively pursued to ensure prompt clarification. Participants’ enrolment will be facilitated by providing clear, concise, and understandable information about the study’s purpose, procedures, benefits, and risks using compelling language and visuals to make the study interesting and engaging. Each participating woman will be offered free cervical cancer screening if desired and will receive an estimated $2.50 credit on their cellphone as an incentive to retain their phone number for the duration of the intervention. We also anticipate some participants’ fatigue and drop-outs during the study’s follow-up period. We will, therefore, mitigate these by training and providing support to the study team on study protocols, including retention techniques such as good communication skills, cultural competence, and specialized knowledge of the target population. The final analysis will be conducted using the intention-to-treat principle. Data will be analyzed using SPSS version 29.0 for Windows. The participants’ characteristics, by intervention arm, will be presented as mean (standard deviation), median (25th—75th centile), and frequency (%) depending on type and distribution. The significance level is set at *P* < 0.05, and all hypothesis tests will be two-sided. We will test the associations between the intervention and HPV vaccination uptake using Pearson’s Chi-square (χ2) or Fisher’s exact test where appropriate. Using multinomial logistic regression analysis, we will adjust for the effects of all possible covariates in this association. In a subgroup analysis of women in the usual care arm, a multivariable binary logistic regression model will be developed using a backward stepwise selection approach to identify factors such as participant’s age, socioeconomic class, parity, marital status, number of GOP clinic attendance, the distance of residence from the clinics, the functionality of cellphones, adolescent girl’s age and school level and other relevant demographic and clinical variables as that are independently associated with HPV vaccination uptake after 6-months of follow-up. Variables associated with vaccination uptake (*P* < 0.10) in the bivariable analyses will be included in the pool of variables for the backward stepwise regression model. An Akaike’s Information Criterion (AIC) will be continually calculated, and the final model step with the lowest AIC will be chosen as the best-fit model. Associations in the final model will be considered significant if *P* < 0.05.

### Ethical considerations

Ethical approval for the “*mHealth-HPVac”* study was obtained from the Health Research Ethics Committee of the Lagos University Teaching Hospital (ADM/DSCST/HREC/APP/6566 – May 10, 2024) and the College of Medicine, University of Lagos (CMUL/HREC/5/24/1464 – May 15, 2024). The purpose and nature of the study will be explained to all potential participants and the willing participant will sign an informed consent form. The trial will adhere to the guidelines outlined in the Standard Protocol Items: Recommendations for Interventional Trials (SPIRIT) checklist for reporting.

#### Quality control and data monitoring

All investigators and research assistants will be required to undergo training including good clinical practice (GCP) training before the trial to guarantee consistent practice. The training will cover a comprehensive understanding of the inclusion/exclusion criteria, follow-up protocols, and questionnaire completion. The research assistant will transfer identifiable data to an electronic database system housed in a secure facility at the trial site. Access to identifiable data will be limited solely to the principal investigator (KSO) throughout and after the trial concludes. The trial will undergo monitoring by quality assurance personnel from the research management office of the College of Medicine, University of Lagos, who will operate independently from the study team. Regular monitoring will occur to ensure the accuracy and quality of data throughout the study duration. Monitors will oversee and verify the essential documents (consent information, enrollment records, protocol deviations, and instances of loss to follow-up) for accuracy and completeness.

### Dissemination of information

The trial will adhere to the reporting guidelines outlined in the Consolidated Standards of Reporting Trials (CONSORT) checklist, and the findings will be published in a peer-reviewed scientific journal. Any significant modifications to the trial protocol will be promptly communicated to the funding agency, study investigators, LUTH and CMUL HREC, trial participants, and the trial registry.

### Study status

At the point of manuscript submission, recruitment for the trial has not yet begun. Enrollment of participants is scheduled to commence in July 2024, with the final participant expected to be included in the trial by September 2024. The anticipated completion date for the trial is March 2025. This manuscript details protocol version 4.0, dated June 6th, 2024.

## Discussion

Cervical cancer, the most common HPV-associated malignancy, is a major public health disease in Nigeria. However, despite the availability of effective vaccines, HPV vaccine uptake remains low in most resource-limited settings including Nigeria [5, 12, 13]. The utilization of mobile technologies has increased significantly in recent years [14] with increased opportunities for mobile health technologies (mHealth) development [15]. Mobile health technology (mHealth) may empower patients to control their health, reduce inequalities [16], and improve the uptake of health interventions such as HPV vaccination. Currently, no study has investigated the use of mobile technologies to enhance HPV vaccination uptake. This protocol, therefore, describes a randomised controlled trial of mHealth technologies using text messages to improve HPV vaccination uptake by evaluating the impact of mobile technologies on HPV vaccination uptake among mothers of unvaccinated girls 9–14 years in Lagos, Nigeria and also determine the factors that affect this vaccination uptake as a means of reducing the disparities in the incidence of cervical cancer through primary prevention facilitated using health promotion to improve HPV vaccination uptake. We have powered the study to detect the primary outcome to show that mHealth can improve HPV vaccine uptake in mothers of unvaccinated girls aged 9–14 years. We believe that if found to be effective, the mHealth intervention strategy may become an important tool for reducing the cervical cancer burden, and its associated morbidity and mortality. This approach could also be utilized for health promotion and prevention efforts targeting other significant diseases in the future. However, a limitation of this trial is that we have not specifically powered to detect the predictors of HPV vaccination uptake in these mothers (although this is a planned secondary outcome). The trial would need to be significantly larger to detect these influencing factors. However, the study will generate preliminary data for hypothesis testing in a future robust and carefully designed prospective cohort study.

### Electronic supplementary material

Below is the link to the electronic supplementary material.

Supplementary Material 1

Supplementary Material 2

Supplementary Material 3

## Data Availability

No datasets were generated or analysed during the current study.
