# Supplementary material for: Impact of mobile health technologies on human papillomavirus vaccination uptake among mothers of unvaccinated girls aged 9–14 years in Lagos, Nigeria (mHealth-HPVac): study protocol of a randomised controlled trial
Source: BMC Cancer. 2024 Jun 20;24:751. doi: 10.1186/s12885-024-12538-6 (PMC11191157; doi:10.1186/s12885-024-12538-6)
Supplement: Supplementary file 2 — Supplementary Material 2 [file 12885_2024_12538_MOESM2_ESM.docx]

**CONSENT DOCUMENT**

**Participant’s Information**

**Impact of mobile health technologies on HPV vaccination uptake among mothers of unvaccinated girls aged 9–14 years in Lagos: A randomized controlled trial (mHealth-HPVac)**

Principal Investigator: **Dr. Kehinde Okunade**

1. **Research introduction:**

We are conducting a study to ascertain the efficacy of an intervention using mobile technologies on Pap smear screening adherence compared to a control condition and also determine the factors affecting the uptake of HPV vaccination among mothers of unvaccinated girls 9–14 years old. You are being requested to participate in this research as a mother to an unvaccinated 9 -14-year-old girl residing in Lagos. This consent form gives you information about the study. You are requested to read it and you are free to ask any questions and they will be answered. After you have understood and if you decide to participate, you will be requested to sign at the bottom.

If you chose to take part in the study, you have the right to withdraw from the study at any time, without having to state a reason.

1. **Background and purpose of study:**

Cervical cancer is a major public health problem and is the fourth most common cancer in women worldwide accounting for an estimated 570,000 new cases annually. More than 80% of the global burden of cervical cancer occurs in less developed regions, where it accounts for almost 12% of all female malignancies. In 2018, an estimated 311,000 deaths were attributed to cervical cancer, which accounts for 7.5% of all female cancer deaths with 70% of these occurring in developing countries. One of the most effective strategies for cervical cancer prevention is vaccination against HPV infection among adolescents before the start of sexual activity. In October 2023, Nigeria embarked on new routine HPV vaccine roll-out campaigns to reach about 7.7 million girls. Under this new immunization protocol, girls aged 9 to 14 years will receive a single dose of the vaccine, which is highly effective at preventing infection of HPV types 16 and 18 that are known to cause at least 70 per cent of cervical cancers. The utilization of mobile technologies has increased significantly in recent years with increased opportunities for mobile health technologies (mHealth) development. The introduction of mHealth may empower patients to control their health, reduce inequalities, and improve the cost-effectiveness of health service delivery.[16] There are only a few reported studies in Sub-Saharan Africa (SSA) that examined the use of mHealth in cancer prevention but there are currently none that have investigated the impact of this intervention on the uptake of HPV vaccination among mothers of eligible unvaccinated school-age girls. This study will, therefore, aim to ascertain the efficacy of an intervention using mobile technologies using short text messages on the uptake of HPV vaccination among mothers of unvaccinated girls 9–14 years old.

1. **Procedures:**

The study involves you participating in a 20 to 30-minute interview with a research assistant. During this interview, the research assistant will ask you questions about yourself and you’ll be asked to consent to providing other information during your subsequent visits to the hospital.

1. **Who will participate in the study?**

Eligible participants are mothers of unvaccinated girls aged 9–14 years; who express willingness to vaccinate their children; own and use a personal cellphone; free from any mental or physical disabilities that inhibit them from understanding the implications of the study and not considering relocating from their current residence within the next year. The exclusion criteria include refusal of consent or withdrawal of consent during the study.

1. **Risks/Discomforts:**

There are no expected physical risks or discomfort from participating in this study.

1. **Benefits:**

You will likely not derive any direct benefit from participating in this study. Once the data is gathered and analyzed, we will use the findings to develop new approaches to improving HPV vaccination adherence. If you would like to discuss any findings from the study, please mention this to the research assistant who will contact you when the study has concluded to share the main findings with you.

1. **Cost:**

Participating in this study will not have any direct cost to you except for your time.

1. **Reimbursement/compensation:**

You will be compensated with an N1,500 credit card for your time and participation in the study.

1. **What are your rights in this study?**

Participation in this study is entirely voluntary. Your subsequent care will not be affected in any way if you participate.

1. **Confidentiality:**

The results of this study will be kept strictly confidential and used only for research purposes. Your name will not appear anywhere on the research findings. Paper records will be kept locked in a secure research office and any electronic documents will be stored securely and only accessed by the research team.

1. **What does your signature mean?**

By signing or putting a thumbprint on the consent form you declare that you have been informed about the research study and are voluntarily agreeing to participate in the survey. Signing or putting a thumbprint means that you understand the information in this consent form. You retain the right to withdraw from the study at any time. You do not waive any of your legal rights by signing this form.

**Consent Certificate**

**Statement of the person obtaining informed consent:**

I have fully explained this research to the respondent and have given sufficient information, including the risks and benefits, to make an informed decision.

Date……...……...……………………… Signature………………………………….

**Statement of the person obtaining informed consent:**

I have read the description of the research. I understand that any participation is voluntary. I know enough about the purpose, methods, risks, and benefits of the research study to judge that I want to take part in it. I have received a copy of this consent form to keep for myself.

Date……...……...……………… Signature/Thumbprint…………………………

For further enquiry, please contact:

1. **Researcher’s contact:**

Dr. K. S. Okunade

Department of Obstetrics & Gynaecology,

College of Medicine, University of Lagos/Lagos University Teaching Hospital,

Mobile: 08034728139

Email: [kehindeokunade@gmail.com](mailto:kehindeokunade@gmail.com)

1. **LUTH Health Research & Ethics Committee’s contact:**

Room 107, Administrative block,

Lagos University Teaching Hospital,

Idi-Araba, Lagos
