# Supplementary material for: Impact of mobile health technologies on human papillomavirus vaccination uptake among mothers of unvaccinated girls aged 9–14 years in Lagos, Nigeria (mHealth-HPVac): study protocol of a randomised controlled trial
Source: BMC Cancer. 2024 Jun 20;24:751. doi: 10.1186/s12885-024-12538-6 (PMC11191157; doi:10.1186/s12885-024-12538-6)
Supplement: Supplementary file 3 — Supplementary Material 3 [file 12885_2024_12538_MOESM3_ESM.docx]

**“mHealth-HPVac” Questionnaire**

Date of enrolment…………………………………………………

Hospital number………………………………………………….

Phone number……………………………………………………

**Section I: Personal Information**

1. Age in years……………………………………………………………………….
2. The number of children……………………………………………………………….
3. Marital status

☐Single

☐Married

☐Widowed

☐Separated

1. Educational level

☐Uneducated

☐Primary school

☐Secondary school

☐Polytechnic/University

☐Postgraduate

1. Occupation

☐Unemployed

☐Housewife

☐Trader

☐Artisan

☐Skilled/Professional

1. Husband/partner’s occupation

☐Unemployed

☐Housewife

☐Trader

☐Artisan

☐Skilled/Professional

1. Tribe

☐Yoruba

☐Ibo

☐Hausa

☐Others

1. The distance of residence from the recruitment site in kilometres (use Google Maps) …………………………….

**Section II: Use of Mobile Phone**

1. The total duration of use of the mobile phone (in years)………………………………
2. The functionality of the mobile phone

☐Fully functional (works all year round)

☐Somehow functional (works at least 6 months in a year)

☐Rarely functional (works at least 3 months in a year)

☐Not functional (never used more than once in a year)

**Section III: Previous Cervical Cancer Screening**

1. Awareness of cervical cancer screening

☐YES

☐NO

1. Previous cervical cancer screening

☐YES

☐NO

1. If YES to Q12, the number of years since the last cervical cancer screening…………………………………….
2. Result of last cervical cancer screening

☐Normal

☐Abnormal

**Section IV: Girl child information**

1. Age of oldest unvaccinated girl child (9–14 years) in years…………………………….
2. School level

☐Year 3

☐Year 4

☐Year 5

☐Year 6

☐Year 7

☐Year 8

☐Year 9

☐Year 10

**Section V: To be completed at the 6-month follow-up.**

1. HPV vaccination of girl child aged 9–14 years.

☐YES

☐NO

1. Date of HPV vaccination…………………………………………………………
2. Number of GOP clinic attendance since enrolment………………………………
3. Was your cellphone fully functional since enrolment (in use for at least 5 months since enrolment)

☐YES

☐NO

**Data collected by: ……………………………………………………………….**

**Signature: …………………………………………………………………………**

**Date: ……………………………………………………………………………….**
